# Macrophages and chemokines as mediators of angiogenesis

**DOI:** 10.3389/fphys.2013.00159

**Published:** 2013-07-05

**Authors:** Jennifer L. Owen, Mansour Mohamadzadeh

**Affiliations:** Department of Infectious Diseases and Pathology, Division of Gastroenterology, Hepatology and Nutrition, Department of Medicine, University of FloridaGainesville, FL, USA

**Keywords:** angiogenesis, chemokines, chemokine receptors, macrophages, tumor-associated macrophages, tumors

## Abstract

Accumulating evidence attests to the important roles of both macrophages and chemokines in angiogenesis. Tumor-associated macrophages or TAMS constitute the major fraction of tumor-infiltrating leukocytes and are recruited by a number of chemoattractants that are produced by the tumor and tumor-associated stroma. This heterogeneous cell population is activated by a variety of stimuli and becomes polarized to result in functionally different phenotypes regarding tumor progression. As opposed to classically activated or M1 macrophages that exhibit anti-tumor functions, most TAMS are considered to be of the alternatively activated or M2 phenotype, and express multiple cytokines, proteases, and chemokines that promote tumor angiogenesis. Chemokines also have disparate effects on angiogenesis regulation, as several members of the CXC and CC chemokine families are potent inducers of angiogenesis, while a subset of CXC chemokines are angiostatic. This review summarizes the current literature regarding the roles and modes of action of macrophage-derived chemokines as mediators of angiogenesis.

## Introduction

Human chemokines are a superfamily of 48 small (approximately 8–14 kDa) chemoattractant cytokines that are divided into four subfamilies, CXC, CC, (X)C, and CX_3_C, based on the arrangement of the conserved N-terminal cysteine residues, where “X” represents any amino acid (Zlotnik and Yoshie, 2000). Systematic chemokine nomenclature is based on the cysteine subfamily, followed by an “L” for ligand and a numerical designation (Zlotnik and Yoshie, 2000; Mukaida and Baba, 2012). Since some chemokines only exist in humans or in mice, human chemokines may be designated with capital letters, while the murine chemokines are written in lower-case letters (Zlotnik and Yoshie, 2012). The biologic effects of these proteins are mediated by a superfamily of 19 seven transmembrane G protein-coupled receptors (7TM GPCRs). There is also a set of “atypical” chemokine receptors that do not mediate cell migration, but rather, regulate inflammation by acting as decoy or scavenger receptors (Mantovani et al., 2010). Several chemokine decoy receptors have been identified (D6, DARC/Duffy antigen receptor for chemokines, CCXCKR); all have mutations that prevent G protein-coupling and thus, intracellular signaling, acting instead to alter local concentrations of chemokines within a microenvironment and influencing subsequent immune responses (Collins et al., 2010; Yoshimura and Oppenheim, 2011).

Chemokines can be thought of as “inflammatory” or “homeostatic” depending on whether they are induced during inflammation or constitutively expressed in certain tissues (Zlotnik et al., 2011). Homeostatic chemokines are expressed in lymphoid or other organs, are involved in leukocyte homing and trafficking, and are well conserved between species (Zlotnik and Yoshie, 2012). Inflammatory chemokines are primarily involved in the recruitment of leukocytes to areas of inflammation and can have marked differences in function between species (Islam et al., 2011; Zlotnik et al., 2011). Inflammatory chemokines play key roles in tumor progression, as they determine the immune cell infiltrate in the tumor microenvironment, modulate the immune response, and participate in angiogenesis and dissemination of the tumor.

In this review, we discuss the potential roles of macrophages and their production of chemokines in modulating angiogenesis.

## Angiogenesis

There are two major processes involved in the formation of blood vessels, vasculogenesis and angiogenesis. Vasculogenesis typically describes the generation of blood vessels *de novo* from mesenchymal blood islands that develop into blood cells and vascular endothelium (Lu et al., 2011). Angiogenesis is defined as the sprouting of new vessels from pre-existing ones (Risau, 1997). Physiologic angiogenesis occurs during embryonic development, wound healing, and female reproductive cycling, and involves vessel destabilization, endothelial cell migration and proliferation, and sprouting. These processes are followed by a resolution phase with reduced endothelial cell proliferation and vessel stabilization (Motz and Coukos, 2011). In the adult organism, angiogenesis is typically associated with pathologic processes such as cancer, stroke, diabetes, and other inflammatory diseases such as psoriasis and arthritis (Kiefer and Siekmann, 2011); unlike physiologic angiogenesis, pathologic angiogenesis does not have a resolution phase and results in a highly disorganized vascular network (Motz and Coukos, 2011). Hypoxia or low oxygen tension is the primary factor in the induction of angiogenesis. Inflammatory cells are recruited to ischemic tissues and extravasate to these areas via tethering to P-selectin expressed on activated endothelial cells and platelets (Egami et al., 2006). Once there, these inflammatory cells release cytokines, vasoactive molecules, and chemokines in response to the hypoxia. The resulting vascular networks of tumors are leaky and hemorrhagic, with abnormal endothelial cell proliferation and apoptosis; they are poorly functional with excessive convoluted branching that results in oxygen depletion and extracellular acidosis (Nagy et al., 2010; Fokas et al., 2012). These chaotic vessels lack distinct venules, capillaries, or arterioles, and are lined by endothelial cells that differ from normal endothelial cells both molecularly and functionally and are supported by abnormal pericytes that are loosely attached and do not provide full coverage to the vessel (Bussolati et al., 2011).

Since they were first isolated from adult peripheral blood in 1997 (Asahara et al., 1997), emerging data have revealed a role for endothelial progenitor cells (EPCs) in the process of tumor neovascularization. Circulating EPCs or angioblasts comprise a very minor subpopulation in the blood that is most likely derived from hemangioblast precursors (Asahara et al., 2011). They were first characterized by their expression of CD31, Flk-1/ vascular endothelial growth factor receptor (VEGFR)-2, Tie-2, and their release of nitric oxide (Asahara et al., 1997; Ahn and Brown, 2009). These progenitor cells home to sites of neovascularization, differentiate into endothelial cells, and have been reported to compose anywhere from <0.01% in B16 melanoma (Purhonen et al., 2008) to >80% of the tumor vasculature in B6RV2 lymphoma (Lyden et al., 2001). Using a preclinical model of murine Lewis lung carcinoma metastasis, investigators found that these cells comprised 12% of the neovasculature in the metastatic lesions, and more importantly, demonstrated that blocking their mobilization significantly inhibited angiogenesis and decreased the formation of lethal macrometastases, implicating these cells in “the angiogenic switch” (Gao et al., 2008). Despite the discrepancies in their reported contributions to the composition of tumor vasculature, these cells can contribute to neovascularization by virtue of their production of pro-angiogenic mediators including VEGF, insulin-like growth factor (IGF)-1, angiopoitin (Ang)-1 and -2, and **CXCL12**/stromal cell-derived factor-1/SDF-1 (Ahn and Brown, 2009).

Vascular endothelial growth factor (VEGF), also known as VEGF-A, is the prototypical pro-angiogenic cytokine secreted by hypoxic tumor cells, tumor-associated macrophages (TAMs), and endothelial cells within the tumor microenvironment. It was originally demonstrated to be an endothelial growth factor and a potent inducer of vascular permeability (Claesson-Welsh and Welsh, 2013). VEGF has also been shown to be chemotactic for monocytes *in vitro* via VEGF receptor 1/FLT1 and VEGFR2/KDR (Murdoch et al., 2008). Thus, this molecule is an obvious target for anti-angiogenic therapy in cancer patients. However, a clinical study using laser capture microdissection (LCM) and gene expression profiling in rectal carcinoma patients using bevacizumab (Genentech), an anti-VEGF antibody, found that CXCL12, CXCR4, and **CXCL6**/granulocyte chemoattractant protein-2/GCP-2 expression were induced in rectal cancer cells with bevacizumab administration, while neuropilin 1 was increased in TAMs (Xu et al., 2009). Furthermore, increased plasma levels of CXCL12 in these patients after treatment were associated with rapid disease progression and metastasis. The authors speculated that the CXCL12–CXCR4 pathway may be a tumor resistance or escape mechanism with anti-VEGF monotherapy, as this pathway is also strongly implicated in angiogenesis (Xu et al., 2009).

## CXC chemokines and the ELR motif

CXC chemokines can be further be classified as ELR^+^ or ELR^−^, based on the presence of a glutamate-leucine-arginine amino acid sequence at the N-terminus of the protein. The **ELR^+^** chemokines, **CXCL1**/growth-regulated oncogene α/GROα, **CXCL2**/GROβ, **CXCL3**/GROγ, **CXCL5**/epithelial cell-derived neutrophil activating peptide-78/ENA-78, **CXCL6**/granulocyte chemoattractant protein-2/GCP-2, **CXCL7**/neutrophil-activating protein-2/NAP-2, and **CXCL8**/ interleukin (IL)-8/IL-8, are chemotactic for neutrophils, and are pro-angiogenic (Raman et al., 2011). All of the murine ELR^+^ CXC chemokines signal via the CXCR2 receptor, while human ELR^+^ CXC chemokines signal primarily through CXCR2, but can also signal through CXCR1 (Keeley et al., 2011). It is important to note that there is no homologue of the human *CXCL8/IL-8* gene in mice or rats (Nomiyama et al., 2010). Additionally, humans have both *CXCR1* and *CXCR2* genes for the ELR^+^ chemokine receptors, whereas *CXCR1* has not been found in mice or rats (Moepps et al., 2006; Mukaida and Baba, 2012). CXCL8 is the prototypic ELR^+^ pro-angiogenic chemokine in its promotion of endothelial cell migration, invasion, and proliferation, all of which result in the formation of capillary-like structures within tumors (Ben-Baruch, 2012). In addition to its secretion by tumor cells, CXCL8 has also been shown to be produced by monocytes when cultured with supernatants from freshly excised breast cancer tissue and mammary tumor cell lines. Moreover, the cultured monocytes also secreted the pro-angiogenic chemokines, CXCL1, CXCL2, CXCL3, CXCL5, and CXCL7, resulting in micro vessel formation, with no production of angiostatic chemokines (Toulza et al., 2005).

CXCL8 may also play a role in angiogenesis as a potent neutrophil chemoattractant (Mantovani et al., 2010; Tazzyman et al., 2013). In multistep pancreatic islet carcinogenesis in RIP1-Tag2 transgenic mice, it was determined that neutrophils were required for the “angiogenic switch” due to their production of matrix metalloproteinase (MMP)-9 that activates VEGF (Nozawa et al., 2006). Using this model, investigators demonstrated that MMP-9 was expressed by neutrophils infiltrating the angiogenic islets and the tumors, while MMP-9-expressing macrophages were located along the periphery of the tumors, where tumor growth and angiogenesis occur (Nozawa et al., 2006). The ELR^+^ chemokine, CXCL6, may also contribute to angiogenesis via its recruitment of neutrophils. For instance, it has been shown that CXCL6, CXCL8, and CCL2 are co-induced in micro vascular endothelial cells after stimulation with pro-inflammatory stimuli (Gijsbers et al., 2005). Using immunohistochemistry (IHC) on patient biopsies, it was shown that endothelial cells from various gastrointestinal tumors (e.g., adenocarcinomas of the esophagus, stomach, colon, and pancreas) expressed CXCL6, which strongly correlated with leukocyte infiltration of the tumors and MMP-9 expression. While CXCL6 only weakly induced the proliferation of endothelial cells, it did synergize with CCL2 in neutrophil chemotaxis, allowing for neutrophil-derived proteases to degrade extracellular matrix and promote neovascularization (Gijsbers et al., 2005). Other mediators implicated in the “angiogenic switch” include, fibroblast growth factor (FGF), PDGFs, lysophosphatic acid (LPA), and angiopoitins (Hanahan and Weinberg, 2011; Fagiani and Christofori, 2013).

The **ELR^−^** chemokines, **CXCL4**/platelet factor-4/PF-4, **CXCL4L1**/CXCL4 variant, **CXCL9**/monokines induced by interferon-γ/Mig, **CXCL10**/interferon-γ inducible protein-10/IP-10, **CXCL11**/IFN-inducible T cell chemoattractant/I-TAC, **CXCL13**/B cell attracting chemokine-1/BCA-1, and **CXCL14**/breast- and kidney-expressed chemokine/BRAK, are chemotactic for lymphocytes and natural killer (NK) cells and are angiostatic (Raman et al., 2011). CXCL4, CXCL4L1, CXCL9, CXCL10, and CXCL11 are all reported to be ligands for CXCR3 (Struyf et al., 2011). These angiostatic chemokines play important roles in tumor progression, as over-expression of CXCL4 inhibits angiogenesis, tumor growth, and metastasis (Yamaguchi et al., 2005). In fact, CXCL4 was the first described angiostatic chemokine, which was found to inhibit endothelial migration and proliferation, and the binding of fibroblast growth factor (FGF)-2 and VEGF to their receptors (Maione et al., 1990; Airoldi and Ribatti, 2011). CXCL4 was once thought to only be expressed by megakaryocytes and platelets, however, human monocytes, mast cells, and activated T cells are now known to secrete this chemokine (Vandercappellen et al., 2011). CXCL4L1 is a homologue of CXCL4 and differs by three amino acid residues at the C-terminus of the protein. Both genes are located on human chromosome 4, and *CXCL4L1*, which is only present in humans and some primates, likely arose from recent duplication of the *CXCL4* gene (Dubrac et al., 2010). These proteins are not identical in function, as CXCL4L1 is a more potent inhibitor of endothelial cell migration and angiogenesis than its homologue, as well as being more diffusible, having a longer half-life, and acting in a paracrine manner, as opposed to CXCL4's juxtacrine activity (Dubrac et al., 2010).

## The CXCL12/CXCR4 axis

An important exception to the **ELR^−^** rule is **CXCL12**/stromal cell-derived factor-1/SDF-1, which is ELR^−^, but promotes angiogenesis via binding to its receptor, CXCR4 (Singh et al., 2013). Currently, CXCR4 is one of the most studied chemokine receptors and is over-expressed in over 20 different human tumors, including prostate, breast, ovarian, lung, pancreatic, colorectal, and melanoma (Balkwill, 2004; Singh et al., 2013). It has been shown that CXCL12 binding to CXCR4 induces Akt phosphorylation and increases production of the major angiogenic factor, VEGF, in the human breast cancer cell line, MDA-MB-23 (Liang et al., 2007). Experimental models of melanoma, colon, pancreatic, thyroid, and prostate cancer have demonstrated that organ directed metastasis is mediated by CXCR4^+^ tumor cells migrating to CXCL12^+^ organs such as the liver and the lungs (Domanska et al., 2013). Studies of glioblastomas and neuroblastomas have also shown that CXCR4^+^ monocytes recruited to tumors promote new vascular formations within the neoplasms; the monocytes first establish themselves within perivascular areas of the tumor and then release pro-angiogenic factors such as VEGF and angiopoitins, with subsequent recruitment of bone marrow-derived endothelial and perivascular progenitors that will compose the vasculature (Jodele et al., 2005; Domanska et al., 2013).

CXCL12 is normally expressed by the mesenchymal stroma of the lungs, liver, lymphatic tissues, and bone marrow (Domanska et al., 2013). Despite its documented role in tumorigenesis, CXCL12 is considered a homeostatic chemokine by virtue of its pivotal role in the retention and homing of hematopoietic stem cells in the bone marrow and in lymphocyte trafficking (Teicher and Fricker, 2010). In addition to its expression on most leukocyte subsets, CXCR4^+^ cells that can directly or indirectly participate in angiogenesis include, smooth muscle cell progenitors, endothelial cell precursors, and immature and mature hematopoietic cells (Petit et al., 2007; Teicher and Fricker, 2010). CXCL12 directly mediates angiogenesis through its binding to CXCR4 on endothelial cells and by recruiting endothelial progenitor cells, while indirectly it induces the secretion of pro-angiogenic factors such as VEGF, CXCL8, and CXCL1 by leukocytes, tumor cells, and endothelial cells that express CXCR4 (Verbeke et al., 2011).

CXCR7 is another receptor with high affinity for CXCL12, and its recent discovery has complicated the understanding of the CXCL12/CXCR4 axis. While CXCR4 and CXCR7 are both moderately expressed on normal endothelial cells, the expression of CXCR7 on endothelial cells within the tumor microenvironment is markedly up-regulated and has recently been suggested as a marker of tumor vasculature in various tumors such as renal carcinoma and gliomas (Singh et al., 2013). Conversely, other researchers demonstrated that co-expression of both CXCR4 and CXCR7 resulted in decreased CXCL12-mediated intravasation of mammary carcinoma cells and fewer metastases of these tumors to the lungs (Hernandez et al., 2011; Singh et al., 2013). Moreover, CXCR7 is also able to bind with low affinity to the angiostatic ELR^−^ chemokine, CXCL11, which itself can also bind to CXCR3 (Lasagni et al., 2003). Clearly, additional research is necessary to better understand the crosstalk between these chemokines and their multiple receptors.

## The lone CX_3_C chemokine

The single member of the CX_3_C subfamily, **CX_3_CL1**/fractalkine, signals through its chemokine receptor, CX_3_CR1, and is unique in that it is a cell surface transmembrane protein that functions as an adhesion molecule that can be proteolytically cleaved by the metalloproteinases, ADAM10 and ADAM 17, to form the active soluble chemoattractant (White and Greaves, 2009). This chemokine recruits lymphocytes, NK cells, and monocytes, and has been shown to participate in angiogenesis through several different mechanisms. For example, Kumar *et al.* have shown that a bone marrow derived CX_3_CR1^+^ monocyte subpopulation is capable of differentiating into smooth muscle-like cells subsequent to CX_3_CL1–CX_3_CR1 interactions in blood vessel walls after vessel injury (Kumar et al., 2010). Furthermore, a competent CX_3_CL1–CX_3_CR1 interaction is necessary for nascent microvessel formation, maturation, and vascular structural integrity in two models of neovascularization, and for the differentiation of CX_3_CR1^+^ monocytes into smooth muscle-like cells *in vivo* (Kumar et al., 2013). Loss of this chemokine/receptor interaction resulted in the development of smaller, leaky, poorly developed, and hemorrhagic microvessels in Matrigel and experimental plaque models of neovascularization (Kumar et al., 2013).

Utilizing inducible fibroblast growth factor receptor 1 (FGFR1) in a murine mammary tumor cell line and its endogenous expression in the breast cancer cell line, HS578T, another laboratory has recently shown that activation of this tyrosine kinase receptor leads to CX_3_CL1 production by tumor cells and subsequently, enhanced macrophage recruitment to mammary cells during the early stages of tumorigenesis *in vitro* and *in vivo* (Reed et al., 2012). By blocking CX_3_CR1 *in vivo*, these researchers demonstrated decreased macrophage infiltration into the mammary epithelium of MMTV-iFGFR1 mice and decreased angiogenesis (Reed et al., 2012). It is important to note that CX_3_CL1 is typically up-regulated on inflamed endothelium via the pro-inflammatory cytokines, IL-1, tumor necrosis factor (TNF-α), and interferon (IFN-γ) (Lee et al., 2013).

As lipid-laden macrophages or “foam cells” are the defining feature of early atherosclerosis, laboratories focusing on this disease process have also studied the molecules that direct monocyte migration from the peripheral blood to vessel walls. Accordingly, Saederup *et al.* created *CX_3_CL1*^−/−^*CCR2*^−/−^*ApoE*^−/−^ mice, and demonstrated independent roles for CCR2 and CX_3_CL1 in the accumulation of macrophages in the artery walls of mice deficient in apolipoprotein E (ApoE) (Saederup et al., 2008). *CCR2* and *CX_3_CR1* are located too close together on murine chromosome 9 for the generation of mice deficient in both receptors. Deletion of both *CX_3_CL1* and *CCR2* resulted in dramatically reduced macrophage accumulation in artery walls compared to deletion of only one of those genes, suggesting that these molecules work additively, and that they recruit different monocyte subsets from the circulating Ly6C^hi^ population in atherogenesis (Saederup et al., 2008; Lee et al., 2013).

## Macrophages

Analogous to the T helper cell Th1/Th2 classification, macrophages can be broadly divided into a classically activated M1 phenotype or alternatively activated M2 macrophages (Hao et al., 2012). M1 pro-inflammatory macrophages are activated by IFN-γ, TNF-α, and engagement of Toll-like receptors (TLRs) by microbial stimuli such as lipopolysaccharide (LPS), and release inflammatory cytokines and reactive oxygen and nitrogen intermediates. These macrophages are typically IL-12^high^, IL-23^high^, and IL-10^low^, promote Th1 responses, are tumoricidal, and can elicit tissue destruction (Mantovani and Sica, 2010; Baay et al., 2011). M2 anti-inflammatory macrophages, on the other hand, are directly induced by interleukin (IL)-4 and IL-13 and indirectly by IL-5, IL-10, IL-21, IL-25, and IL-33 (Liu and Yang, 2013). The chemokines, CCL2, **CCL17**/thymus and activation-regulated chemokine/TARC, and **CCL22**/macrophage-derived chemokine/MDC have also been shown to promote M2 polarization of macrophages (Mantovani and Sica, 2010). These cells are usually IL-12^low^, IL-23^low^, and IL-10^high^, and are involved in immunosuppression, tissue repair (including angiogenesis), and tumor promotion (Mantovani and Sica, 2010; Baay et al., 2011).

**Figure 1:**
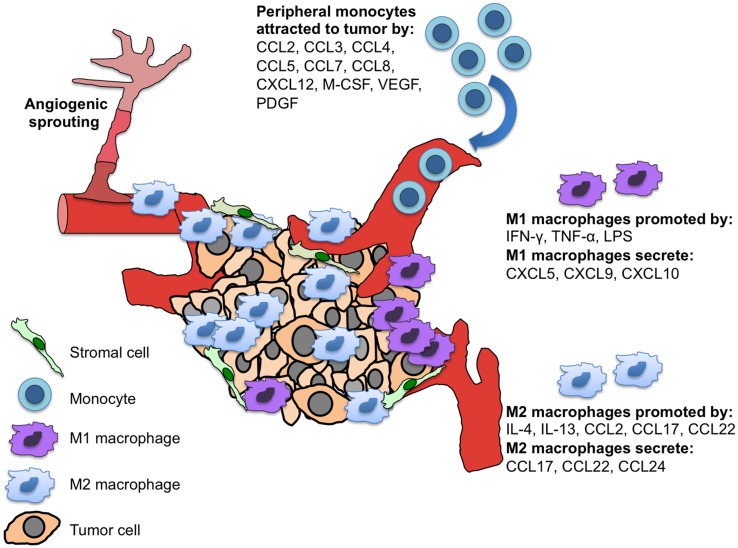
**M1 and M2 macrophages and their differential chemokine secretion in the tumor microenvironment.** Tumor and tumor-associated stromal cells secrete chemokines and cytokines to recruit monocytes to the site of the tumor. These peripheral monocytes differentially develop into polarized tissue macrophages (M1 vs. M2), which have distinct chemokine secretion patterns and functions within the tumor.

M1 and M2 macrophages also express different chemokines; M1 cells produce pro-inflammatory CXCL5/epithelial cell-derived neutrophil-activating factor-78/ENA-78, CXCL9/monokine induced by IFN-γ/MIG, and CXCL10/IFN-γ-inducible protein-10/IP-10, while M2 macrophages make CCL17, CCL22, and **CCL24**/eotaxin-2 (Figure 1) (Mantovani et al., 2004; Traves et al., 2012). Further classification of alternatively activated macrophages into M2a, M2b, and M2c has also been suggested (Gordon and Martinez, 2010). Typically, tumor associated macrophages (TAMs) are M2 macrophages and play important roles in angiogenesis, metastasis, and the generation of immunosuppressive regulatory T cells (Tregs) (Mantovani et al., 2009).

## Tumor-associated macrophages (TAMs)

The induction of angiogenesis is considered to be a “hallmark” of cancer, a distinctive capability that is necessary for tumor growth and dissemination (Hanahan and Weinberg, 2011). While angiogenesis was once thought occur after tumor cells acquired an invasive phenotype, it is now appreciated that this event occurs early in tumorigenesis during pre-malignant lesions of the breast, prostate, gastrointestinal tract, cervix, uterus, lung, and squamous cell carcinoma of the head and neck (Raica et al., 2009). Furthermore, it has been shown that TAMs play a key role in the induction of angiogenesis, and their infiltration precedes vascular remodeling in the PyMT (mammary epithelial cell restricted expression of the polyoma middle T oncoprotein) mouse model of breast cancer (Lin et al., 2006). Depletion of macrophages in this tumor model using mice carrying the homozygous null allele (*Csf1^op^*) for the monocyte growth factor, colony stimulating factor (CSF)-1, caused a 50% reduction in vascular density and resulted in delayed tumor progression and metastasis (Lin et al., 2006; Murdoch et al., 2008).

Tumor-promoting inflammation is considered to be the seventh “hallmark” of cancer (Hanahan and Weinberg, 2011). The microenvironment of solid tumors comprises many other non-malignant cell types, including the cells of blood and lymphatic vessels, fibroblasts, adipocytes, and leukocytes such as macrophages, dendritic cells, lymphocytes, neutrophils, eosinophils, mast cells, and myeloid-derived immune suppressor cells (MDSCs), which are characterized by co-expression of the macrophage surface marker, CDllb, and the neutrophil surface marker, Gr1. TAMs are the most prominent component of the leukocyte infiltrate within tumors, and one meta-analysis found that intratumoral macrophage density correlated with a poor patient prognosis in over 80% of studies (Bingle et al., 2002; Halin et al., 2009). Macrophages are a heterogeneous population of leukocytes that play many important roles in immune regulation, angiogenesis, tumor progression, and metastasis. Accumulating data suggest that peripheral blood monocytes extravasate into tumors and differentiate into tissue macrophages, accumulating in distinct tumor microenvironments depending on chemokine expression pattern (Murdoch et al., 2008; Lee et al., 2013). This accumulation of macrophages occurs within hypoxic areas of the neoplasm that contain necrotic tissue and is mediated primarily by the CC chemokine, **CCL2**/monocyte chemoattractant protein-1/MCP-1 (Murdoch et al., 2008). CSF-1, VEGF, placental growth factor (PGF), CXCL12, CXCL8, and MMP-9 have also been reported to be involved in the mobilization and recruitment of hematopoietic cells from the bone marrow to the sites of tumors (De Palma et al., 2007).

## CC chemokines

CC chemokines are chemotactic for monocytes, dendritic cells (DCs), eosinophils, basophils, lymphocytes, and NK cells (Zlotnik and Yoshie, 2012). Multiple studies have demonstrated a correlation between the levels of the inflammatory chemokines, CCL2 and CCL5, and the number of myeloid cells within tumors (Soria and Ben-Baruch, 2008; Allavena et al., 2011). The primary monocyte-recruiting chemokine, CCL2, regulates the trafficking of monocytes, macrophages, and other inflammatory cells by binding to its receptor, CCR2 (Zhang et al., 2010). CCL2 expression has been demonstrated in many types of cancer including, multiple myeloma, melanoma, esophageal, gastric, colorectal, lung, breast, ovary, and prostate cancer (Craig and Loberg, 2006; Zhang et al., 2010). CCL2 indirectly contributes to angiogenesis by attracting TAMS, which secrete pro-angiogenic cytokines such as VEGF, platelet-derived growth factor (PDGF), transforming growth factor (TGF)-β, and CXCL8/IL-8, and the proteolytic enzymes, MMP-2 and MMP-9 (Mantovani et al., 2010). CCL2 can also directly induce angiogenesis in endothelial cells, which express its receptor, CCR2 (Salcedo et al., 2000), and induce VEGF and hypoxic-inducible factor (HIF)-1 in tumor cells (Zhang et al., 2010).

Other tumor-derived chemotactic factors secreted by both malignant and stromal cells that attract peripheral monocytes to the site of tumors include **CCL3**/macrophage inflammatory protein-1α/MIP-1α, **CCL4**/macrophage inflammatory protein-1β/MIP-1β, **CCL5**/regulated on activation normal T cell expressed and secreted/RANTES, **CCL7**/MCP-3, **CCL8**/MCP-2, CXCL12, and the cytokines, macrophage colony stimulating factor (M-CSF), PGF, and VEGF (Figure 1) (Lewis and Pollard, 2006; Murdoch et al., 2008; Mukaida and Baba, 2012). After macrophages have successfully migrated into the hypoxic region of the tumor, their movements become restricted by decreased expression of CCR2 and CCR5 via hypoxia-mediated down-regulation of these receptors (Sica et al., 2000; Bosco et al., 2004; Lee et al., 2013). The authors' of these studies speculated that this may be a mechanism to retain recruited macrophages at hypoxic sites; and together with the observation that hypoxia mediates up-regulation of CXCR4 within macrophages and tumor cells (Schioppa et al., 2003), is suggestive of plasticity in chemokine receptor expression within hypoxic tissues (Bosco et al., 2004).

Interestingly, investigators have revealed another connection in the angiogenesis nexus involving macrophages and CCL2. The transcription factor, Twist 1, has previously been shown to play multiple roles in tumor initiation and progression, including induction of the epithelial mesenchymal transition (EMT) and degradation of the extracellular matrix (Yang et al., 2004), with increased expression having a positive correlation with metastasis and poor survival in several aggressive human tumors, including breast and colorectal cancer (Gomez et al., 2011). Via, in part, to repression of E-cadherin transcription, the epithelial mesenchymal transition permits carcinoma cells to migrate away from the site of the primary tumor through the lymphatics and/or peripheral blood to form metastatic tumor foci (Qin et al., 2012). Low-Marchelli *et al.* recently demonstrated an additional and novel tumor-promoting role for this transcription factor in the induction of CCL2 production by human and murine mammary tumor cells, which serves to recruit infiltrating CCR2^+^ macrophages and to induce angiogenesis (Low-Marchelli et al., 2013). These authors suggested that induction of CCL2 by Twist 1 in tumor cells recruits TAMs that then promote extravasation and metastatic seeding in other organs by virtue of the production of MMPs, which can degrade the extracellular matrix, release matrix-bound growth factors, and allow endothelial cells to invade the tumor during angiogenesis (Low-Marchelli et al., 2013).

## A “chemokine-like” factor

A cytokine that is not a true chemokine but is considered to be “chemokine-like” is macrophage migration inhibitory factor (MIF), which a non-cognate ligand for both CXCR2 and CXCR4. The name of this cytokine may not be completely accurate, as studies have shown a role for MIF in the recruitment of monocytes in the pathogeneses of arthritis and glomerulonephritis (Bernhagen et al., 2007). Furthermore, it has been demonstrated that MIF is a critical molecule in vascular processes, and its expression is up-regulated in endothelial cells, smooth muscle cells, and macrophages during the development of atherosclerotic lesions in mice and humans (Bernhagen et al., 2007). Recently, investigators have also found that hypoxia-induced MIF recruits endothelial progenitor cells (EPCs) in a CXCR4^+^dependent manner, suggesting a possible role for MIF in angiogenesis (Simons et al., 2011).

## Concluding remarks

Chemokines affect multiple signaling pathways of inflammatory diseases and tumor initiation and development including, cellular proliferation, survival and apoptosis, leukocyte recruitment, cellular migration/metastasis, and of course, angiogenesis. The newly appreciated complexity of the crosstalk between the chemokines, CXCL11 and CXCL12, and their receptors CXCR3, CXCR4, and CXCR7, illustrate the importance of additional studies in order to better understand the opposing and synergistic effects of pleiotropic chemokines and their promiscuous chemokine receptors. Like chemokines, macrophages also play multiple roles in inflammatory diseases and tumor progression. Thus, identifying the mechanisms by which macrophages are recruited to sites of inflammation or tumor, and exactly how these leukocytes influence angiogenesis may lead to better targeted therapeutic applications in patients with cancer and other inflammatory diseases involving vascular pathology.

### Conflict of interest statement

The authors declare that the research was conducted in the absence of any commercial or financial relationships that could be construed as a potential conflict of interest.
